# A Review of the Current Landscape of SARS-CoV-2 Main Protease Inhibitors: Have We Hit the Bullseye Yet?

**DOI:** 10.3390/ijms23010259

**Published:** 2021-12-27

**Authors:** Guillem Macip, Pol Garcia-Segura, Júlia Mestres-Truyol, Bryan Saldivar-Espinoza, Gerard Pujadas, Santiago Garcia-Vallvé

**Affiliations:** Research Group in Cheminformatics & Nutrition, Departament de Bioquímica i Biotecnologia, Campus Sescelades, Universitat Rovira i Virgili, 43007 Tarragona, Catalonia, Spain; guillem.macip@gmail.com (G.M.); polgarse2@gmail.com (P.G.-S.); juliamt110@gmail.com (J.M.-T.); bsaldivar.emc2@gmail.com (B.S.-E.)

**Keywords:** COVID-19, M-pro inhibitors, 3CL-pro inhibitors, computational chemistry, protease inhibitors, virtual screening

## Abstract

In this review, we collected 1765 severe acute respiratory syndrome coronavirus 2 (SARS-CoV-2) M-pro inhibitors from the bibliography and other sources, such as the COVID Moonshot project and the ChEMBL database. This set of inhibitors includes only those compounds whose inhibitory capacity, mainly expressed as the half-maximal inhibitory concentration (IC_50_) value, against M-pro from SARS-CoV-2 has been determined. Several covalent warheads are used to treat covalent and non-covalent inhibitors separately. Chemical space, the variation of the IC_50_ inhibitory activity when measured by different methods or laboratories, and the influence of 1,4-dithiothreitol (DTT) are discussed. When available, we have collected the values of inhibition of viral replication measured with a cellular antiviral assay and expressed as half maximal effective concentration (EC_50_) values, and their possible relationship to inhibitory potency against M-pro is analyzed. Finally, the most potent covalent and non-covalent inhibitors that simultaneously inhibit the SARS-CoV-2 M-pro and the virus replication in vitro are discussed.

## 1. Introduction

**Figure 1:**
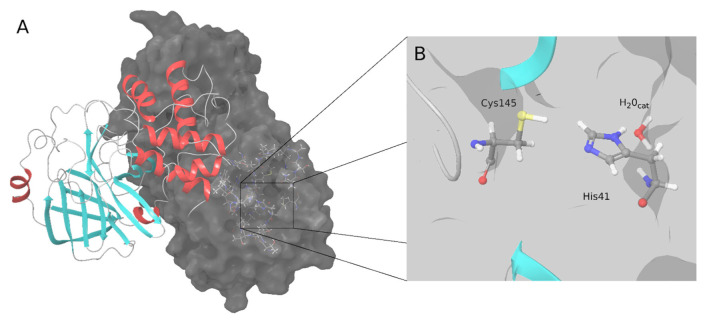
SARS-CoV-2 M-pro structure. (**A**) Biological assembly of the M-pro in its dimeric form. The left protomer is shown in cartoon representation, colored by protein secondary structure, and the right protomer is displayed as a surface. (**B**) Detailed snapshot of the catalytic water, Cys145 and His41.

Since the onset of the COVID-19 pandemic, the scientific community has focused on studying the severe acute respiratory syndrome coronavirus 2 (SARS-CoV-2) virus that causes the disease and on developing therapies and vaccines, several of which have been developed in record time. In the pharmacological field, no drugs have yet been definitively approved to inhibit the replication of SARS-CoV-2 and stop the development of COVID-19. Several targets are being studied, including the main protease (M-pro), which plays an essential role in virus replication [1]. This protease and the papain-like protease cleave the pp1a and pp1ab polyproteins to produce several nonstructural proteins, including M-pro itself, required for virus replication and transcription [1]. The high conservation of M-pro among related viruses, the importance of M-pro in the replication of the virus and the fact that M-pro only exists in coronaviruses and not in humans makes it an attractive target for the development of antiviral drugs [2,3,4]. SARS-CoV-2 M-pro has 306 amino acids that form three domains (I, II and III) [4]. The M-pro binding site is located between domains I and II, and domain III is involved in dimerization, which is essential for M-pro activity [4]. Similar to the M-pro from SARS-CoV-1 and other coronaviruses, SARS-CoV-2 M-pro has two catalytic amino acids, His41 and Cys145 (Figure 1). A catalytic water molecule is also important and makes a strong hydrogen bond with His41 [5]. Although some allosteric binding sites have been identified for the SARS-CoV-2 M-pro [6,7,8,9], most of the inhibitors crystallized within the M-pro bind to the active site [10]. One strategy used to find SARS-CoV-2 M-pro inhibitors, especially at the beginning of the pandemic, was drug repositioning [2,11,12,13]. This strategy is based on looking for drugs approved for one disease (therefore, its safety and possible adverse effects are known) that can be used to treat another—in this case, COVID-19. One of the most widely used computational tools for repositioning drugs, or looking for compounds with new activities, is what is known as protein-ligand docking. This tool predicts whether a particular molecule can bind (and, if it can, how) to a particular target (for example, the SARS-CoV-2 M-pro [14]). However, protein-ligand docking has several limitations, such as the consideration of the protein as a rigid body and the lack of confidence in the ability of scoring functions to give accurate binding energies [15,16]. In addition, the flexibility of the SARS-CoV-2 M-pro makes it a challenging target for small-molecule inhibitor design [17]. Using two different SARS-CoV-2 M-pro structures and five protein-ligand docking methods, we have recently shown that docking scores or the Gibbs free energy (∆G) calculated with an MM-GBSA method [18] do not correlate with bioactivity [19], probably because of the inability of common docking programs to correctly reproduce the binding modes of SARS-CoV-2 M-pro inhibitors [20]. This reinforces the idea that it is essential to validate the results obtained by protein-ligand docking or any other computational tool, especially when analyzing SARS-CoV-2 M-pro inhibitors [19,21,22,23]. The results of protein-ligand docking can be computationally validated by re-docking, cross-docking and applying the same protocol to a set of known active compounds and a set of decoy or inactive compounds [19]. Protein-ligand docking is expected to discriminate decoys from active compounds. If docking scores are used to rank the potency of a set of compounds, it must first be demonstrated that there is a correlation between docking scores and activity or potency, for example, expressed as IC_50_ values [19]. However, the best way to validate the predictions of protein-ligand docking is to experimentally test the predicted bioactivity of selected hits.

Since the beginning of the COVID-19 pandemic, developing SARS-CoV-2 M-pro inhibitors has been an active area of research. However, it did not have to start from scratch. Previous research about protease inhibitors, especially from SARS-CoV and MERS-CoV, proved to be useful [24,25]. Known inhibitors of proteases from HIV and Hepatitis C virus, in addition to calpain and caspase-3 inhibitors, were systematically analyzed to test their capacity to inhibit the SARS-CoV-2 M-pro [26]. Compounds developed against the M-pro of other coronaviruses were also tested, and some were found to be potent SARS-CoV-2 M-pro inhibitors [24,27,28]. The complete genome sequence of SARS-CoV-2 [29] and the first crystallized structure of the SARS-CoV-2 M-pro [4] were two important milestones in the development of new SARS-CoV-2 M-pro inhibitors. The article describing the first crystallized SARS-CoV-2 M-pro structure (the 6LU7 structure) also presented the first SARS-CoV-2 M-pro inhibitors [4]. These first inhibitors included the N3 compound, which had previously been developed as a protease inhibitor for multiple coronaviruses, including SARS-CoV and MERS-CoV, approved drugs (such as disulfiram and carmofur) and preclinical or clinical-trial drug candidates (ebselen, shikonin, tideglusib, PX-12 and TDZD-8) [4]. Since then, thousands of compounds have been suggested as SARS-CoV-2 M-pro inhibitors through computational methods such as protein-ligand docking, high-throughput screening experiments, computer-aided design and synthesis of new compounds. Several articles have reviewed the SARS-CoV-2 M-pro inhibitors discovered to date [25,28,30,31,32,33,34,35,36,37,38]. In this review, we collected 1765 SARS-CoV-2 M-pro inhibitors from the bibliography and other sources such as the COVID Moonshot project [39,40] and the ChEMBL release 29 database [41]. This set of inhibitors includes only those compounds whose inhibitory capacity, mainly expressed as the half-maximal inhibitory concentration (IC_50_) value, against M-pro from SARS-CoV-2 has been determined. We did not include compounds predicted only by docking or other computational tools. As we have discussed previously, to avoid false positives and false negatives, the results of automated protein-ligand docking should not be used as the only evidence to predict SARS-CoV-2 M-pro inhibitors [19]. Several covalent warheads are used to treat covalent and non-covalent inhibitors separately. Chemical space, the variation of the IC_50_ inhibitory activity when measured by different methods or laboratories and the influence of 1,4-dithiothreitol (DTT) are discussed. When available, virus replication inhibition values, measured with a cellular antiviral assay, were collected, and their relationship with the inhibitory potency against M-pro is shown. Finally, the most potent inhibitors that simultaneously inhibit the SARS-CoV-2 M-pro and the virus replication in vitro are discussed.

## 2. SARS-CoV-2 M-Pro Inhibitors

**Figure 2:**
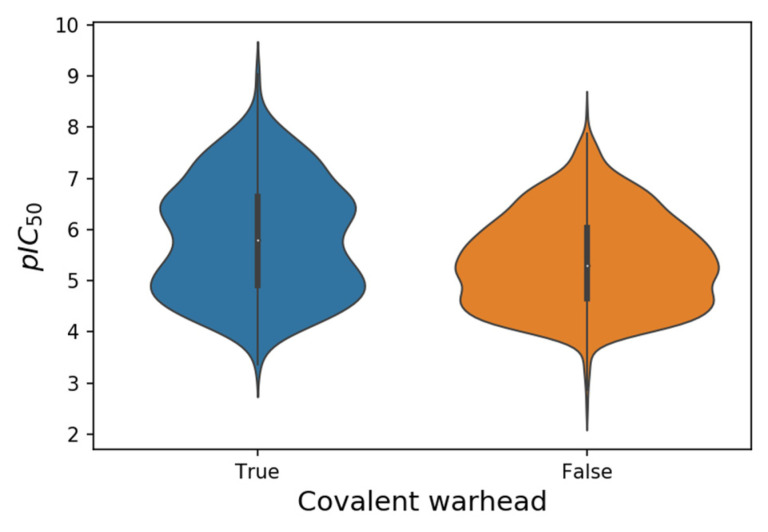
Violin plots of the pIC_50_ values from 552 putative covalent and 1213 non-covalent SARS-CoV-2 M-pro inhibitors.

Table 1 shows the origin of the non-redundant set of 1765 SARS-CoV-2 M-pro inhibitors collected (see supplementary file S1). This set of inhibitors includes only those compounds whose inhibitory capacity, mainly expressed as the IC_50_ value, against M-pro from SARS-CoV-2 has been determined. A total of 758 compounds were extracted from peer-reviewed articles published between January 2020 and August 2021. When multiple IC_50_ values were found for the same compound, the mean value was calculated. From a set of 1037 M-pro inhibitors with an IC_50_ value downloaded from the COVID Moonshot project [39,40] on 1st October 2021, the compounds that had already been collected from the bibliographic search were discarded. In the end, 999 compounds were collected from COVID Moonshot. The IC_50_ values of these compounds were estimated as the mean value of the IC_50_ values from two biochemical assays: a fluorescence-based assay and a RapidFire Mass Spectrometry assay. Finally, 8 compounds were collected from the ChEMBL database [41], which contained more SARS-CoV-2 M-pro inhibitors, but most of them had already been collected from the bibliography. The SMILES of the 1765 SARS-CoV-2 M-pro inhibitors were standardized with the Standardizer 21.15.0 program from ChemAxon (http://www.chemaxon.com, accessed on 4 September 2021). The pIC_50_ values of the SARS-CoV-2 M-pro inhibitors collected range from 2.5 to 9.0 (Table 1). Putative covalent inhibitors were identified by the presence of typical covalent warheads (Table 2). When one of these warheads is in the appropriate position within the M-pro binding site, it can form a covalent bond, usually with the catalytic Cys145 [25]. There are twice as many non-covalent inhibitors as putative covalent inhibitors (Table 1), although pIC_50_ values are highest in some putative covalent inhibitors (Table 1 and Figure 2). However, conventional IC_50_ measurements are of limited value for characterizing the potency of irreversible covalent inhibitors, because incubation for different periods of time would provide different IC_50_ values [42]. Other parameters, such as molecular weight, LogP, number of hydrogen bond donors and hydrogen bond acceptors were similar between the covalent and non-covalent sets (see Supplementary Figure S1).

**Figure 3:**
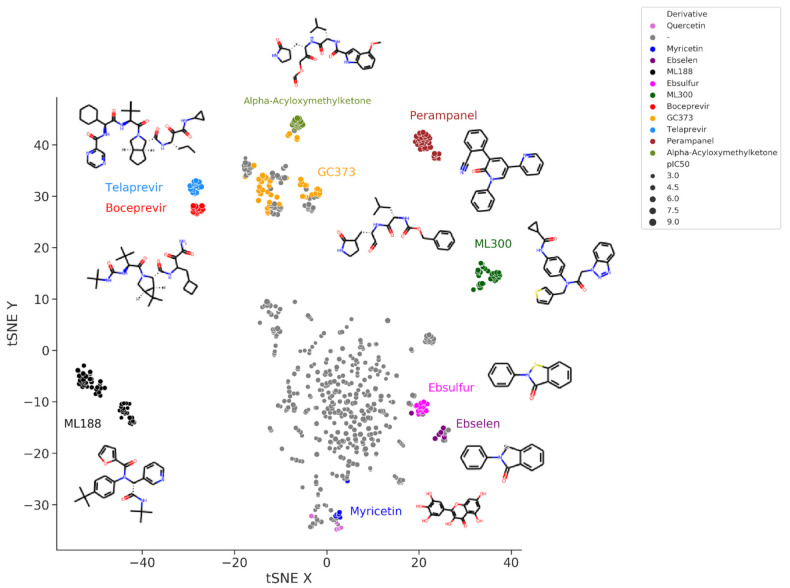
t-Distributed Stochastic Neighbor Embedding (t-SNE) visualization of the chemical space of a set of SARS-CoV-2 M-pro inhibitors extracted from the bibliography. Embedding is based on the 2048-bit Morgan fingerprint. Markers are colored according to several manually attributed chemotypes.

Figure 3 shows the t-Distributed Stochastic Neighbor Embedding (t-SNE) visualization of the chemical space of the set of SARS-CoV-2 M-pro inhibitors extracted from the bibliography. In this representation, more similar compounds are closer together. Peptidomimetic compounds, such as alpha-acyloxymethylketones, telaprevir, boceprevir, GC373 and their derivatives, which mimic natural peptide substrates, are closer together at the top left of the figure. Other clusters of compounds represent derivative compounds that have been synthesized from a lead compound to increase its bioactivity. Thus, derivatives from perampanel, ML300, ML188, ebsulfur, ebselen and myricetin form well-defined clusters. Perampanel derivatives are an example of a very successful increase in activity. Perampanel was first predicted as a SARS-CoV-2 M-pro inhibitor by consensus docking [2]. This prediction was confirmed by Jorgensen and coworkers, although perampanel showed only an approximate IC_50_ of 100–250 μM [43]. The same authors also optimized this compound and synthesized several derivative compounds [44,45,46]. Some of these perampanel derivatives have IC_50_ values in the low nanomolar range and are some of the most potent non-covalent SARS-CoV-2 M-pro inhibitors found to date.

ML300 and ML188 are non-covalent inhibitors that were developed against the M-pro from SARS-CoV-1 [47,48]. Both compounds have been used to obtain more potent SARS-CoV-2 M-pro inhibitors that can inhibit SARS-CoV-2 replication in infected cells [49,50]. Boceprevir and telaprevir are approved protease inhibitors for treating hepatitis caused by the hepatitis C virus. Both compounds have been identified several times as covalent inhibitors of the SARS-CoV-2 M-pro [43,51,52,53,54,55,56,57,58]. New bicycloproline derivatives have been designed and synthesized from them both [59]. All compounds inhibited SARS-CoV-2 M-pro in vitro, with IC_50_ values ranging from 7.6 to 748.5 nM [59]. In addition, two of them, MI-09 and MI-30, showed excellent antiviral activity in a cell-based assay and significantly reduced lung viral loads and lesions in a transgenic mouse model of SARS-CoV-2 infection [59]. GC376 is a covalent M-pro inhibitor that was developed as an inhibitor of the main protease of the feline coronavirus FCoV [60] that also showed activity against the M-pro from MERS and SARS-CoV viruses [61]. Its IC_50_ activity against SARS-CoV-2 M-pro ranges between 0.026 and 0.89 μM [51,52,61,62,63,64,65,66,67]. GC376 is a prodrug, and its bisulphite adduct is converted to an aldehyde to form GC373. This aldehyde forms a covalent interaction with the catalytic Cys145 of the SARS-CoV-2 M-pro [61]. Several GC373 and GC376 derivative compounds have been designed and assayed [63,68,69]. Some of them, such as UAWJ248 [70], are more potent than GC376. A group of peptidomimetic compounds with an alpha-acyloxymethyl ketone warhead designed to form an irreversible covalent bond with Cys145 showed IC_50_ values against the SARS-CoV-2 M-pro in the nM range [71]. They also inhibited SARS-CoV-2 replication and presented low cytotoxicity and good stability [71]. Ebselen is a covalent inhibitor of the SARS-CoV-2 M-pro, although its specificity has been questioned [72,73]. Several derivative compounds of ebselen and its sulfur derivative ebsulfur have been analyzed [74,75]. Some of the derivative compounds displayed more potent M-pro inhibition than ebselen and ebsulfur [74,75]. However, the promiscuous behavior of ebselen and ebsulfur and their lack of cellular antiviral activity [74,75] may also be applied to their derivatives. Myricetin is a flavonoid that acts as a non-peptidomimetic and covalent inhibitor of SARS-CoV-2 [76,77]. Its covalent behavior was unexpected and caused by the pyrogallol moiety that formed a covalent bond with Cys145 [76]. Myricetin and its derivatives inhibit SARS-CoV-2 M-pro and SARS-CoV-2 replication in cells [76,77,78,79], and form a cluster at the bottom of Figure 3, near quercetin and other flavonoids.

### 2.1. Activity Assays for Identifying SARS-CoV-2 M-Pro Inhibitors

Several methods have been developed or adapted for quantifying the potency of the SARS-CoV-2 M-pro inhibitors [73,80,81,82,83,84,85,86,87,88,89]. These methods demonstrate the mechanism of action of antiviral drugs and do not require cells infected with SARS-CoV-2 or a laboratory with biosafety level 3 containment facilities. When combined with high-throughput sample processing and analysis, hundreds or thousands of compounds can be screened. These methods use a marked substrate, usually a peptide derivative, and when the M-pro is present, the substrate is cut, which induces the emission of a signal, usually fluorescence. In the presence of an M-pro inhibitor, signal intensity is reduced, and the potency of the inhibitor can be quantified. Some methods consist of an in vitro screen and use a purified M-pro protein, but they do not account for cell permeability, metabolization or cytotoxicity and cannot be used to accurately predict the cellular activity of M-pro inhibitors [73,83]. The M-pro protein can be expressed by transforming a bacterium with a plasmid encoding the SARS-CoV-2 M-pro. For purification purposes, an M-pro modified with the addition of specific residues, known as a tag, to the N- or C-terminus of the protein can be used. However, the tag may interfere with the binding of M-pro to its ligands [90]. M-pro requires a native N-terminus to form the active dimer [70,91], so a C-terminal His-tag has been used. However, this C-terminal His-tag can lower the binding affinity of a given ligand [90]. To overcome the limitations of the in vitro screens, cell-based assays have been developed [73,80,83,84,87,88]. In these assays, the cells express the M-pro and the reporter used and can differentiate cytotoxicity from true M-pro inhibition [83]. However, these assays often require a biosafety level 2 laboratory. To complement the activity assays, a thermal shift binding assay can be performed. This assay is based on the thermal stabilization of a protein when it binds to a protein. A ΔTm shift of up to 18 °C has been observed for some SARS-CoV-2 inhibitors [51,70,73,92,93]. However, the thermal shift assay might not be suitable for analyzing non-covalent M-pro inhibitors [90]. For covalent inhibitors, the binding can be confirmed by native mass spectrometry.

**Figure 4:**
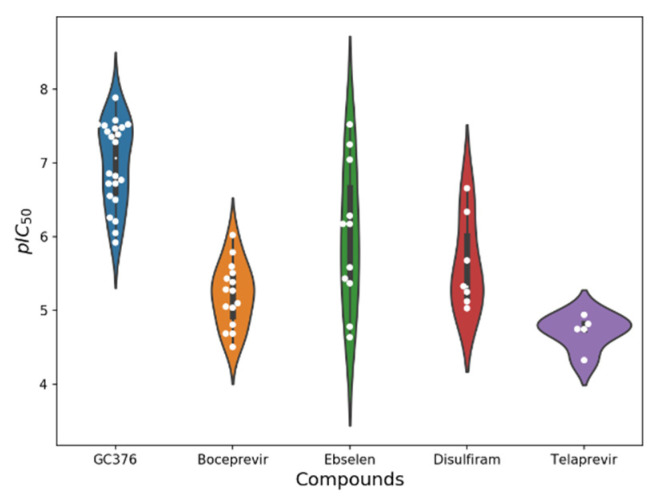
Comparison of multiple pIC_50_ values calculated by different laboratories. The pIC_50_ values of the five most evaluated compounds, GC376, boceprevir, ebselen, disulfiram and telaprevir, are shown as white points to highlight that different laboratories and methods can estimate different pIC_50_ values.

In all assays for identifying SARS-CoV-2 M-pro inhibitors, negative and positive controls are needed. As a positive control, a known SARS-CoV-2 M-pro inhibitor is used. Some of the inhibitors most commonly used as positive controls are GC376, boceprevir, ebselen, disulfiram and telaprevir. After the inhibition of the SARS-CoV-2 M-pro has been detected, a dose-response assay can be used to calculate the IC_50_ or its derivative pIC_50_. Lower values of IC_50_ and higher values of pIC_50_ represent more potent inhibitors. Comparisons between IC_50_ values obtained with different methods and by different laboratories must be made with great care. Furthermore, irreversible covalent M-pro inhibitors cannot be unambiguously ranked for potency using IC_50_ values [94]. To show the differences in the pIC_50_ estimations for the same compound, we identified the compounds with the highest number of pIC_50_ values from our dataset of SARS-CoV-2 M-pro inhibitors. Figure 4 shows the variation in the pIC50 values of the five most evaluated compounds, GC376, boceprevir, ebselen, disulfiram and telaprevir. These compounds are usually used as positive controls, and multiple pIC_50_ values for each compound have been calculated. The variation in the pIC_50_ values for three of these five compounds can be higher than two pIC_50_ units. This means that the estimations of the IC_50_ values of a compound could differ by a factor of 100 between two different laboratories or methods.

**Figure 5:**
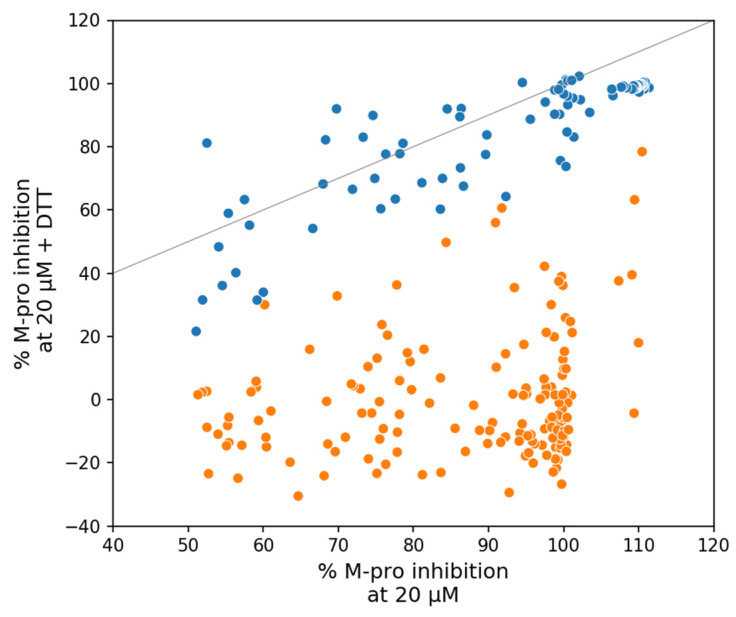
Effect of 1mM of DTT on M-pro inhibition in a group of 246 SARS-CoV-2 M-pro inhibitors [77]. A gray line represents the points where the presence of DTT does not affect the % of inhibition. Compounds far below the gray line are DTT-sensitive inhibitors and are colored orange. Compounds near the gray line are DTT insensitive inhibitors and are colored blue. The R^2^ value of the insensitive compounds is 0.75 (*p* = 1.1 × 10^−27^).

The inhibitory activity achieved in enzymatic assays is sensitive to the method and conditions used [73,95]. Compounds such indinavir, lopinavir, nelfinavir, saquinavir, and tipranavir have shown no M-pro inhibitory activity at some of the concentrations analyzed [54,55,62,96]. Other compounds, such as candesartan, chloroquine, dipyridamole, montelukast and oxytetracycline, did not inhibit M-pro in four different assays tested [73]. The presence of DTT has been reported to affect the inhibitory activity of M-pro covalent inhibitors, as it maintains M-pro in a reduced state and eliminates non-specific thiol-reactive compounds [51,91]. Thus, if the inhibitory effect of an M-pro inhibitor is eliminated or greatly reduced by the presence of DTT, then the inhibition is non-specific. Therefore, the enzymatic inhibition potency of cysteine protease inhibitors measured in the absence of DTT should not be used to predict cellular antiviral activity [72]. Some of the SARS-CoV-2 M-pro inhibitors whose inhibitory activity is eliminated or reduced by the presence of DTT are carmofur, disulfiram, ebselen, tideglusib, shikonin, PX-12 [72,73,97], and zinc pyrithione [77]. The results of a thermal shift-binding assay in the presence of DTT showed that some of these compounds (i.e., disulfiram, ebselen, tideglusib, shikonin and PX-12) did not bind to SARS-CoV-2 M-pro [72]. This means that these compounds are promiscuous cysteine inhibitors that are not specific for M-pro [72,73]. Figure 5 shows the effect of 1mM of DTT on the M-pro inhibition in a group of 246 SARS-CoV-2 M-pro inhibitors [77]. A total of 156 of these compounds showed a relative reduction in SARS-CoV-2 M-pro inhibition of more than 30% and can be considered as “DTT sensitive” [77].

### 2.2. Activity Assays for Identifying Molecules That Inhibit SARS-CoV-2 Replication

**Figure 6:**
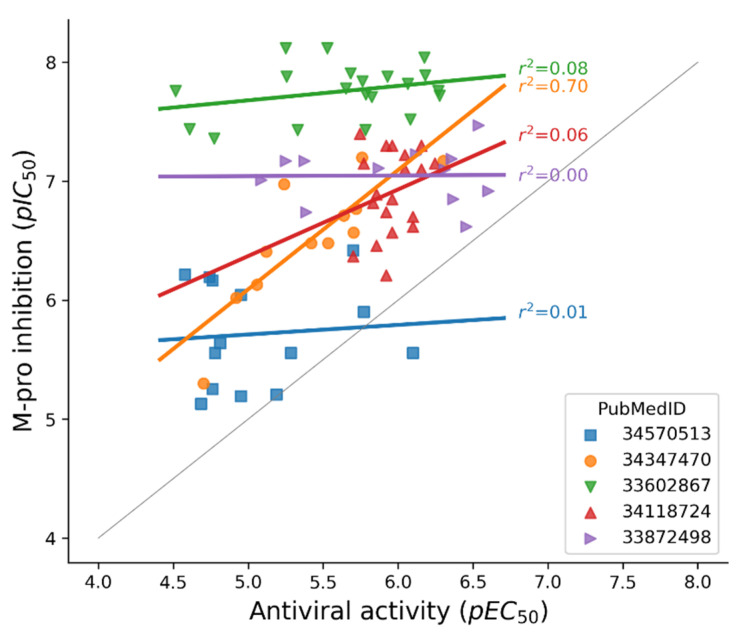
Comparison between the pIC_50_ values of some SARS-CoV-2 M-pro inhibitors and their antiviral activities, measured as pEC_50_ values. To avoid comparisons between values from different laboratories, data from five articles that calculate the pIC_50_ and pEC_50_ for a set of compounds are shown independently. The PubMedID of the articles is indicated. A gray line represents the diagonal where pIC_50_ and pEC_50_ values are equal.

In addition to testing the inhibition of SARS-CoV-2 M-pro in vitro or in a cell-based assay, the ability of a compound to inhibit the SARS-CoV-2 replication also needs to be tested. An antiviral assay, using cells infected with SARS-CoV-2, is the gold standard assay, although it requires a biosafety level 3 laboratory. The Vero E6 cell line is one of the most common cell lines used for this analysis, but other cell lines have also been used. However, it has been reported that Vero cells express high levels of some efflux transporters, which may mask the true activity of some compounds [93]. A dose-response assay can be used to calculate a half maximal effective concentration (EC_50_) value, defined as the concentration of the compound that reduces the viral-induced cytopathic effect by 50% (with respect to the virus control). However, the cytotoxicity of the compounds needs to be estimated to discount that the observed effect is not due to the toxicity of the compounds. For this purpose, the half-maximal cytotoxic concentration (CC_50_) is usually used. Not all the compounds that inhibit the SARS-CoV-2 M-pro in vitro can inhibit the SARS-CoV-2 replication. For example, carmofur, disulfiram, ebselen, PX-2, shikonin and tideglusib cannot inhibit SARS-CoV-2 replication in cell cultures [64,72,73]. It has been suggested that the possible antiviral activity of lopinavir and nelfinavir is due to their cytotoxicity [83,98]. The antiviral activity of other compounds against SARS-CoV-2, measured as an EC_50_ value, is slightly more than 10 times their M-pro inhibitory activity, measured as an IC_50_ value [54]. One possible explanation why compounds with high inhibitory activity in an in vitro M-pro assay show little or no activity in a cell assay is their low lipophilicity and the resulting poor cell membrane permeability. Figure 6 compares the pIC_50_ values of some SARS-CoV-2 M-pro inhibitors with their anti SARS-CoV-2 activities, measured as pEC_50_ values. To avoid comparisons between values from different laboratories, data from five articles [49,59,69,99,100] that calculate the pIC_50_ and pEC_50_ for a set of compounds are shown independently. In all but one case, the pEC_50_ values are lower than the pIC_50_ values, showing that the potency of a compound to inhibit SARS-CoV-2 replication in cells cannot always be inferred from the potency to inhibit the M-pro determined in vitro.

### 2.3. Most Potent Covalent and Non-Covalent Inhibitors of SARS-CoV-2 M-Pro

**Figure 7:**
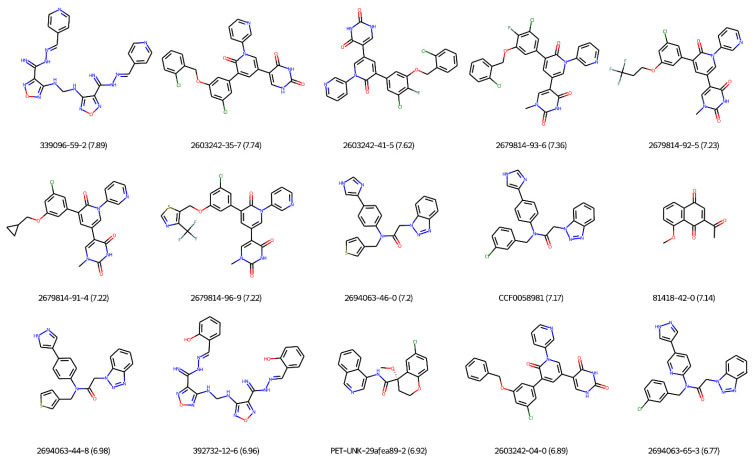
The 15 most potent non-covalent inhibitors of the SARS-CoV-2 M-pro. Only M-pro inhibitors with pIC_50_ values that can inhibit SARS-CoV-2 replication in a cellular antiviral assay are shown. pIC_50_ values are shown in parentheses.

Figure 7 and Table 3 show the 15 most potent non-covalent inhibitors of the SARS-CoV-2 M-pro. Only M-pro inhibitors that have pIC_50_ values and can inhibit SARS-CoV-2 replication in a cellular antiviral assay were included. The compound with the CAS number 339096-59-2, also named compound M3 [101], was obtained from the optimization of a previous SARS-CoV-2 M-pro inhibitor, named compound 13 [102]. Compound 339096-59-2 inhibited the SARS-CoV-2 M-pro in vitro with an IC_50_ of 13 nM and displayed anti-SARS-CoV-2 activity in Vero-E6 cells infected with SARS-CoV-2 [101]. The EC_50_ value was 16 nM [101]. This compound also inhibited in vitro human TMPRSS2 and furin enzymes, which are required for viral entry [101]. Compounds with the CAS number 2603242-35-7, 2603242-41-5, 2679814-93-6, 2679814-92-5, 2679814-91-4, 2679814-96-9, 2603242-04-0 are perampanel derivatives that can inhibit the SARS-CoV-2 M-pro in vitro, with IC_50_ values between 0.128 and 0.018 μM [44,46]. The most potent compounds in this set, 2603242-35-7 and 2603242-41-5, showed antiviral activity in a viral plaque assay but lacked antiviral activity in a 3-(4,5-dimethylthiazol-2-yl)-2,5-diphenyltetrazolium bromide (MTT) assay [44] (Table 3). In addition, both compounds showed the highest cytotoxicity [44]. More promising are the results of the other perampanel derivatives. In particular, 2679814-93-6 showed lower values of EC_50_ and negligible cytotoxicity in Vero E6 cells [44]. Compounds 2694063-46-0, CCF0058981, 2694063-44-8 and 2694063-65-3 are derived from ML300 with IC_50_ values against SARS-CoV-2 M-pro of 0.063, 0.068, 0.106 and 0.171 μM, respectively [49]. CCF0058981 showed the highest anti-SARS-CoV-2 activity in infected Vero E6 cells, with an EC_50_ of 497 nM, and low cytotoxicity (CC_50_ > 50 μM) [49]. The compound with the CAS number 81418-42-0 is a juglone derivative that exhibited an IC_50_ value of 72 nM against the SARS-CoV-2 M-pro and that effectively suppressed the replication of SARS-CoV-2 in Vero E6 cells [103]. Compound 392732-12-6 (MCULE-7013373725–0) is a SARS-CoV-2 M-pro inhibitor, with an IC_50_ value of 0.11 μM that also showed significant inhibition activity against SARS-CoV-2 replication (EC_50_ = 0.11 µM) [102]. Interestingly, this compound also inhibited the SARS-CoV-2 papain protease (PL-pro) and human furin protease [102]. The dual activity against the viral and host proteases and dual activity against SARS-CoV-2 M-pro and PL-pro are very interesting. However, more studies about the cytotoxicity of this compound are needed [102]. Its selectivity index (i.e., the ratio between the antiviral activity and cytotoxicity) has room for improvement [102]. PET-UNK-29afea89-2 is a 3-aminopyridine compound from the COVID Moonshot project submitted in October 2020. It showed a high SARS-CoV-2 inhibition and anti-SARS-CoV-2 activity, with IC_50_ and EC_50_ values of 0.129 and 0.244 μM, respectively [40]. However, it was revealed to be a metabolically unstable compound [40]. The attempts to improve the stability of the compound slightly decreased compound potency but significantly increased metabolic stability and oral bioavailability [40].

**Figure 8:**
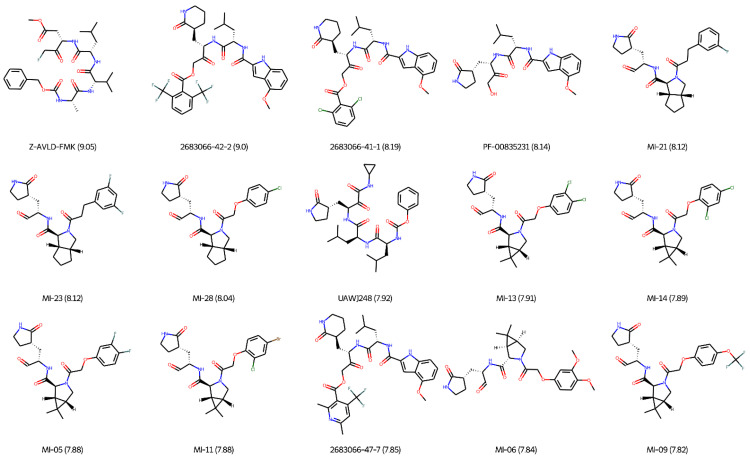
The 15 most potent covalent inhibitors of the SARS-CoV-2 M-pro. Only M-pro inhibitors with pIC_50_ values that can inhibit the SARS-CoV-2 replication in a cellular antiviral assay are shown. pIC_50_ values are shown in parentheses.

Some of the covalent inhibitors of the SARS-CoV-2 M-pro are more potent than the non-covalent inhibitors. Table 4 and Figure 8 show the 15 most potent covalent inhibitors of the SARS-CoV-2 M-pro. Only M-pro inhibitors that have pIC_50_ values and can inhibit SARS-CoV-2 replication in a cellular antiviral assay were included. Z-AVLD-FMK is a peptidomimetic fluoromethylketone (FMK) inhibitor that was improved from a caspase inhibitor to mimic the natural substrates of the SARS-CoV-2 M-pro [97]. It has the highest inhibitory potency of all SARS-CoV-2 M-pro inhibitors, with an IC_50_ value of 0.9 nM [97]. It also inhibited the SARS-CoV-2 replication in Vero E6 cells, with an EC_50_ value of 66 μM [97]. Although Z-AVLD-FMK showed no toxicity at the concentrations assayed, FMKs may present host cell toxicity [97]. Z-AVLD-FMK could be used as a starting point for developing effective antiviral drugs against SARS-CoV-2 [97]. Compounds 2683066-42-2, 2683066-41-1 and 2683066-47-7 are peptidomimetic with an alpha-acyloxymethylketone warhead and a six-membered lactam glutamine mimic [71]. They are potent SARS-CoV-2 M-pro inhibitors with IC_50_ values of 1, 6.4 and 14 nM, respectively [71]. They inhibited SARS-CoV-2 replication in Vero E6 cells and showed low cytotoxicity and good plasma and glutathione stability [71]. Compound 2683066-42-2 also displayed selectivity for SARS-CoV-2 M-pro over several cathepsines [71,93]. More advanced compounds based on alpha-acyloxymethylketone should improve metabolic stability [71]. PF-00835231 is a peptidomimetic compound with a hydroxymethylketone warhead that was a development candidate for SARS-CoV-1, but the end of the SARS-CoV-1 outbreak suspended its development [104]. It is one of the most potent SARS-CoV-2 M-pro inhibitors with an IC_50_ value between 5.8 and 8 nM [104,105,106] and shows high selectivity over human proteases [93]. It exhibited potent activity against SARS-CoV-2 and other coronaviruses as a single agent [93,104]. Furthermore, PF-00835231 has synergistic activity in combination with remdesivir [93]. Pfizer has started a clinical trial of PF-07304814 (also known as lufotrelvir), a prodrug that is metabolized to PF-00835231 [93]. However, PF-07304814 must be administered by intravenous infusion, and other orally active candidates, such as PF-07321332, are better for clinical development [38]. PF-07321332 has recently been described as a SARS-CoV-2 M-pro inhibitor with in vitro pan-human coronavirus antiviral activity, excellent off-target selectivity and in vivo safety profiles [107]. It forms a reversible covalent bond with the M-pro Cys145 through a nitrile substituent [107]. Furthermore, it has demonstrated oral activity in a mouse-adapted SARS-CoV-2 model and has achieved oral plasma concentrations exceeding the in vitro antiviral cell potency in a phase I clinical trial in healthy human participants [107]. Currently, PF-07321332 is in phase 3 trials, and it could be the first approved M-pro inhibitor to be used to treat SARS-CoV-2. MI-21, MI-23, MI-28, MI-13, MI-14, MI-05, MI-11, MI-06 and MI-09 are derivatives of boceprevir or telaprevir with IC_50_ values against the SARS-CoV-2 M-pro ranging between 7.6 and 15.2 nM [59] (Table 4). All of these compounds are aldehyde-based inhibitors and showed anti-SARS-CoV-2 activity in Vero E6 cells with EC_50_ values ranging from 0.66 to 5.63 μM [59]. MI-30 showed an even better EC_50_ value of 0.54 nM, although its IC_50_ value (17.2 nM) is slightly higher than the previously described boceprevir or telaprevir derivatives [59]. The two compounds that showed the best anti-SARS-CoV-2 activity, MI-09 and MI-30, also improved SARS-CoV-2 induced lung lesions in a transgenic mouse model of SARS-CoV-2 infection and displayed good pharmacokinetic properties in rats [59]. UAWJ248 is a GC376 analog with an alpha-ketoamide warhead that binds irreversibly to the Cys145 from SARS-CoV-2 M-pro [70]. This compound was designed on the basis of the x-ray crystal structure of SARS-CoV-2 Mpro with GC-376 (with PDB code 6WTT), to satisfy the side-chain preferences of S1′, S2, S3, and S4 M-pro pockets [70]. UAWJ248 inhibited the SARS-CoV-2 M-pro with an IC_50_ value of 12 nM [108]. However, its anti-SARS-CoV-2 potency was lower, showing an EC_50_ of 20.49 μM against infected Vero E6 cells (Table 4) [70].

## 3. Conclusions

Since the beginning of the COVID-19 pandemic, the scientific community has tried to find a drug to inhibit the SARS-CoV-2 life cycle. The SARS-CoV-2 M-pro enzyme has been extensively studied, and its inhibitors are promising effective drugs for fighting against SARS-CoV-2. The first attempts to discover SARS-CoV-2 M-pro inhibitors used previously developed protease inhibitors or tried to repurpose drugs from other diseases. Neither attempt was entirely effective, due to, among other things, the flexibility of the SARS-CoV-2 M-pro and the inability of protein-docking methods to predict the binding modes and potency of SARS-CoV-2 M-pro inhibitors. Several in vitro and in cellulo (using live cells) methods have been developed to measure the inhibitory potency of a compound against the SARS-CoV-2 M-pro. In vitro methods need to express and purify SARS-CoV-2 M-pro, so some tags are sometimes added. However, especially if they are located at the N-terminus, these tags can interfere with the binding of M-pro to its ligands. The activity values obtained by different laboratories or with different methods or conditions must be compared with great care. The presence of DTT has been reported to affect the inhibitory activity of covalent M-pro inhibitors. If the inhibitory effect of an M-pro inhibitor is eliminated or greatly reduced by the presence of DTT, the inhibition is not specific. Therefore, the potency of inhibition measured in the absence of DTT should not be used by itself. The potency of a compound to inhibit SARS-CoV-2 replication in cells cannot always be inferred from the potency to inhibit M-pro, determined in vitro. An antiviral assay that uses cells infected with SARS-CoV-2 provides a better estimate of the potency of a compound to inhibit virus replication. However, if it is to be ruled out that the toxicity of the compounds is responsible for the antiviral activity, the cytotoxicity of the compounds needs to be determined.

In this review, we collected 1765 SARS-CoV-2 M-pro inhibitors. This set of compounds could be useful to validate a virtual screening procedure. The search for common covalent warheads identifies putative covalent inhibitors. Although we have not yet hit the bullseye and no drug has yet been approved to inhibit M-pro, we may be close. Improving derivatives of a leading compound has proven to be a very successful strategy for finding potent SARS-CoV-2 M-pro inhibitors. Some derivative compounds designed in less than two years since the start of the COVID-19 pandemic represent an important step toward the development of new anti–SARS-CoV-2 drugs. Currently, there are several compounds with low nanomolar IC_50_ values against SARS-CoV-2 M-pro and high anti-SARS-CoV-2 efficacy in cell models, with values comparable to those of the FDA-approved RNA polymerase inhibitor remdesivir. We hope that a SARS-CoV-2 M-pro inhibitor will be approved soon, so we can add a new tool to fight against SARS-CoV-2 or future coronavirus pandemics.

## Figures and Tables

**Table 1:** Number of SARS-CoV-2 M-pro inhibitors collected.

SARS-CoV-2 M-Pro Inhibitor Set	Number of Compounds (Covalent/Non-Covalent) ^1^	pIC_50_ Range	pIC_50_ Range Covalent	pIC_50_ Range Non-Covalent
From the bibliography	758 (346/412)	2.5–9.0	3.4–9.0	2.5–8.3
From COVID Moonshot	999 (205/794)	4.0–7.8	4.0–7.8	4.0–7.4
From ChEMBL	8 (1/7)	5.4–6.1	5.4	5.5–6.1
All	1765 (552/1213)	2.5–9.0	3.4–9.0	2.5–8.3

^1^ Putative covalent and non-covalent inhibitors were identified by the presence or absence of the covalent warheads shown in Table 2.

**Table 2:** Covalent warheads that can be used to identify putative covalent inhibitors. It shows the SMARTS that can be used to identify each warhead and some examples of SARS-CoV-2 inhibitors that contain each warhead. These covalent warheads were used to identify putative covalent inhibitors among the known SARS-CoV-2 M-pro inhibitors.

Warhead	SMARTS	Examples
Acrylamide	[C;H2:1]=[C;H1]C(N)=O	CVD-0004255
Chloroacetamide	Cl[C;H2:1]C(N)=O	BFC204
Vinylsulfonamide	NS(=O)([C;H1]=[C;H2:1])=O	
Nitrile	N#[C:1]-[*]	Isavuconazole
Michael acceptors	C=!@CC=[O,S]	Cinanserin, MPI2, MPI9, N3
Alpha-ketoamide	C(=O)(C=O)N	Boceprevir, narlaprevir, telaprevir, UAWJ248
Aldehyde	[CX3H1](=O)	GC373, MI-05, MI-06, MI-09, MI-11, MI-13, MI-14, MI-21, MI-23, MI-28
Bisulfite adduct of aldehyde	C(O)S(=[OX1])([O])(=[OX1])	GC376
Urea carbonyl	[NX3][CX3](=[OX1])([NX3,nX3])	Carmofur
Bis(dialkylaminethiocarbonyl)disulfide	[CX3](=[SX1])SS[CX3](=[SX1])	Disulfiram
Carbamoylsulfanyl	[NX3,nX3][C,c](=[OX1])([SX2,sx2])	Tideglusib
Disulfide	[SX2][SX2]	PX-12
Hydroxymethyl ketone	[CX3H0](=[OX1])[CH2][OH]	PF-00835231
Alkoxymethyl ketone	[CX3H0](=[OX1])[CH2][OX2H0]	2683066-41-1, 2683066-42-2, 2683066-47-7
Acyloxymethyl ketone	[CX3H0](=[OX1])[CH2][OX2H0][CX3H0](=O)	2683066-41-1, 2683066-42-2, 2683066-47-7
Fluoro, Chloro-methyl ketone	[CX3H0](=[OX1])[CH2][Cl,F]	Z-AVLD-FMK
Ebselen related	[Se]n(c=O)	Ebselen

**Table 3:** Data from the 15 most potent non-covalent inhibitors of the SARS-CoV-2 M-pro.

Compound	IC_50_ μM (pIC_50_)	EC_50_ μM	CC_50_ μM	Reference
339096-59-2 (M3)	0.013 (7.89)	0.016 ^a^	Higher than the EC_50_ by 2.3-fold	[101]
2603242-35-7 (21)	0.018 (7.74)	11.30 ^a,b^	1.7 ^a^	[44]
2603242-41-5 (23; 18)	0.024 (7.62)	0.84 ^a,b^	1.15 ^a^	[44,46]
2679814-93-6 (19)	0.044 (7.36)	0.08 ^a^	>32.5 ^a^	[46]
2679814-92-5 (17)	0.059 (7.23)	0.82 ^a^	>100 ^a^	[46]
2679814-91-4 (16)	0.061 (7.22)	1.20 ^a^	82 ^a^	[46]
2679814-96-9 (21)	0.061 (7.22)	1.08 ^a^	>100 ^a^	[46]
2694063-46-0 (21)	0.063 (7.20)	1.74 ^a^	-	[49]
CCF0058981 (41)	0.068 (7.17)	0.50 ^a^	>50 ^a^	[49]
81418-42-0 (15)	0.072 (7.14)	4.55 ^a^	viability ^a^ >90% at conc. <= 20 μM	[103]
2694063-44-8 (19)	0.106 (6.98)	5.76 ^a^	-	[49]
392732-12-6 (13)	0.11 (6.96)	0.11 ^a^	0.41 ^c^	[102]
PET-UNK-29afea89-2	0.129 (6.92)	0.244 ^d^	lack of toxicity ^d^	[40]
2603242-04-0 (14)	0.128 (6.89)	3.20 ^a^	12.3 ^a^	[44]
2694063-65-3 (40)	0.171 (6.77)	1.91 ^a^	-	[49]

^a^ In Vero E6 cells. ^b^ Lacked antiviral activity in an MTT assay. ^c^ In mammalian cells. ^d^ In Calu-3 cell line.

**Table 4:** Data from the 15 most potent covalent inhibitors of the SARS-CoV-2 M-pro.

Compound	IC_50_ μM (pIC_50_)	EC_50_ μM	CC_50_ μM	Reference
Z-AVLD-FMK	0.0009 (9.05)	66.0 ^a^	-	[97]
2683066-42-2 (15h)	0.001 (9.0)	0.16 ^a^	>200	[71]
2683066-41-1 (15g)	0.0064 (8.19)	0.52 ^a^	>200	[71]
PF-00835231 (4)	0.0057–0.008 0.0072 ^b^ (8.14)	88.9 ^a^	>100 ^a^	[93,104,105,106]
MI-21	0.0076 (8.12)	2.97 ^a^	>500 ^a^	[59]
MI-23	0.0076 (8.12)	5.63 ^a^	>500 ^a^	[59]
MI-28	0.0092 (8.04)	0.67 ^a^	>500 ^a^	[59]
UAWJ248	0.012 (7.92)	20.49 ^a^	>250 ^a^	[70]
MI-13	0.0124 (7.91)	2.08	>500 ^a^	[59]
MI-14	0.0130 (7.89)	0.66 ^a^	>500 ^a^	[59]
MI-05	0.0132 (7.88)	5.57 ^a^	>500 ^a^	[59]
MI-11	0.0133 (7.88)	1.18 ^a^	>500 ^a^	[59]
2683066-47-7 (15m)	0.014 (7.85)	0.47 ^a^	>200	[71]
MI-06	0.0145 (7.84)	1.73 ^a^	>500 ^a^	[59]
MI-09	0.0152 (7.82)	0.86 ^a^	>500 ^a^	[59]

^a^ In Vero E6 cells. ^b^ Mean value of multiple IC_50_.

## Data Availability

All data generated and analyzed during this study are included in this published article and its supplementary information files or are available from the corresponding author on reasonable request.

## Supplementary Materials

The following are available online at https://www.mdpi.com/article/10.3390/ijms23010259/s1.
