# Supplementary figures and images for: A Review of the Current Landscape of SARS-CoV-2 Main Protease Inhibitors: Have We Hit the Bullseye Yet?
Source: Int J Mol Sci. 2021 Dec 27;23(1):259. doi: 10.3390/ijms23010259 (PMC8745775; doi:10.3390/ijms23010259)

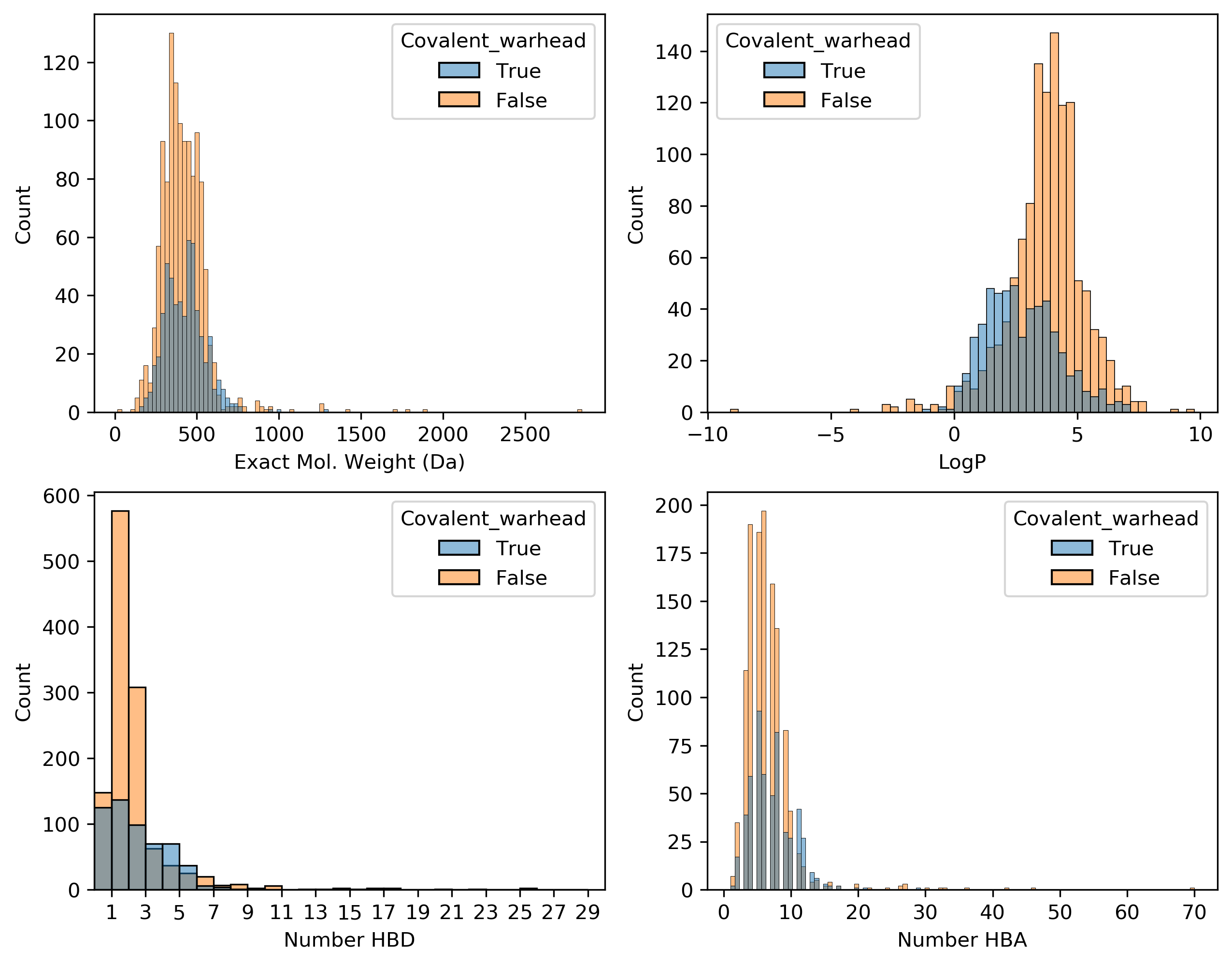

Supplement: Supplementary file 1 [file ijms-23-00259-s001.zip › Fig_S1.tiff]
